# Comparative Proteomics Identifies Host Immune System Proteins Affected by Infection with *Mycobacterium bovis*

**DOI:** 10.1371/journal.pntd.0004541

**Published:** 2016-03-30

**Authors:** Vladimir López, Margarita Villar, João Queirós, Joaquín Vicente, Lourdes Mateos-Hernández, Iratxe Díez-Delgado, Marinela Contreras, Paulo C. Alves, Pilar Alberdi, Christian Gortázar, José de la Fuente

**Affiliations:** 1 SaBio. Instituto de Investigación en Recursos Cinegéticos IREC CSIC-UCLM-JCCM, Ciudad Real, Spain; 2 CIBIO/InBIO, Centro de Investigação em Biodiversidade e Recursos Genéticos, Universidade do Porto, Campus Agrário de Vairão, Vairão, Portugal; 3 Departamento de Biologia, Faculdade de Ciências da Universidade do Porto (FCUP), Porto, Portugal; 4 Departamento de Sanidad Animal, Facultad de Veterinaria, Universidad Complutense de Madrid, Madrid, Spain; 5 Wildlife Biology Program, University of Montana, Missoula, Montana, United States of America; 6 Department of Veterinary Pathobiology, Center for Veterinary Health Sciences, Oklahoma State University, Stillwater, Oklahoma, United States of America; Swiss Tropical and Public Health Institute, SWITZERLAND

## Abstract

Mycobacteria of the *Mycobacterium tuberculosis* complex (MTBC) greatly impact human and animal health worldwide. The mycobacterial life cycle is complex, and the mechanisms resulting in pathogen infection and survival in host cells are not fully understood. Eurasian wild boar (*Sus scrofa*) are natural reservoir hosts for MTBC and a model for mycobacterial infection and tuberculosis (TB). In the wild boar TB model, mycobacterial infection affects the expression of innate and adaptive immune response genes in mandibular lymph nodes and oropharyngeal tonsils, and biomarkers have been proposed as correlates with resistance to natural infection. However, the mechanisms used by mycobacteria to manipulate host immune response are not fully characterized. Our hypothesis is that the immune system proteins under-represented in infected animals, when compared to uninfected controls, are used by mycobacteria to guarantee pathogen infection and transmission. To address this hypothesis, a comparative proteomics approach was used to compare host response between uninfected (TB-) and *M*. *bovis*-infected young (TB+) and adult animals with different infection status [TB lesions localized in the head (TB+) or affecting multiple organs (TB++)]. The results identified host immune system proteins that play an important role in host response to mycobacteria. Calcium binding protein A9, Heme peroxidase, Lactotransferrin, Cathelicidin and Peptidoglycan-recognition protein were under-represented in TB+ animals when compared to uninfected TB- controls, but protein levels were higher as infection progressed in TB++ animals when compared to TB- and/or TB+ adult wild boar. MHCI was the only protein over-represented in TB+ adult wild boar when compared to uninfected TB- controls. The results reported here suggest that *M*. *bovis* manipulates host immune response by reducing the production of immune system proteins. However, as infection progresses, wild boar immune response recovers to limit pathogen multiplication and promote survival, facilitating pathogen transmission.

## Author Summary

Mycobacteria of the *Mycobacterium tuberculosis* complex (MTBC) are zoonotic pathogens representing a serious health problem for humans and animals worldwide. The life cycle of mycobacteria is complex, and the mechanisms resulting in pathogen infection and survival in host cells are not fully understood. Eurasian wild boar are natural reservoir hosts for MTBC and a model for mycobacterial infections and tuberculosis. The results of this study broaden our understanding of the molecular epidemiology of zoonotic tuberculosis and fill important gaps in knowledge of this topic. The results suggested that mycobacteria manipulate host immune response by reducing the production of immune system proteins. However, as infection progresses, wild boar immune response recovers to limit pathogen multiplication and promote survival, facilitating pathogen transmission. As previously reported in other obligate intracellular bacteria, host-mycobacteria interactions probably reflect a co-evolutionary process in which pathogens evolved mechanisms to subvert host response to establish infection, but hosts also evolved mechanisms to limit pathogen infection and promote survival. Subsequently, mycobacteria benefit from host survival by increasing the probability for transmission to continue their life cycle. These results provide relevant information to develop tools to evaluate risks for tuberculosis caused by MTBC and for disease control in humans and animals.

## Introduction

Tuberculosis (TB) caused by mycobacteria of the *Mycobacterium tuberculosis* complex (MTBC) is one of the world's most common causes of death from infectious diseases [1]. Animal TB is caused by infection with *Mycobacterium bovis* and closely related members of the MTBC. Cattle are the main health concern regarding animal TB in industrialized countries, but other mammalian species are also infected with mycobacteria of the MTBC [2,3]. Additionally, human TB cases due to *M*. *bovis* infection are reported every year [4,5]. Eurasian wild boar (*Sus scrofa*) are natural reservoir hosts for MTBC in some regions and therefore vaccination strategies are being developed for TB control in this species [6–9]. Additionally, wild boar are a model for mycobacterial infection and TB reproducing some of the clinical characteristics observed in human cases such as lung pathology and latent infection [9,10].

The life cycle of mycobacteria is complex and not fully characterized [11]. It is generally accepted that after inhalation into the lung or entry to the oropharyngeal cavity, the principal entry routes, mycobacteria of the MTBC are phagocytized by macrophages. As with other intracellular bacteria, mycobacteria survive inside macrophages by escaping host immune response, which results in the formation of a granuloma that effectively contains infected cells. A change in the host-bacterial equilibrium of granulomas is thought to result in the release of infected cells outside containment and onward transmission of mycobacteria to susceptible hosts [11]. In wild boar, mycobacterial infection occurs mostly through oral-nasal routes and mandibular lymph nodes are the most frequently affected tissue by the formation of granulomatous lesions, and the main organ responsible for infection dissemination within the organism [12]. However, generalized infection affects lungs, therefore increasing the risk for pathogen transmission through the oral-nasal route [13]. In the wild boar TB model, mycobacterial infections affect the expression of innate and adaptive immune response genes in mandibular lymph nodes and oropharyngeal tonsils, and Complement component 3 (C3) and Methylmalonyl-CoA mutase (MUT) have been proposed as correlates with resistance to natural mycobacterial infection [10, 14–16]. However, the mechanisms used by mycobacteria to manipulate host immune response are still not fully understood.

Our hypothesis is that the immune system proteins under-represented in infected animals when compared to uninfected controls are used by mycobacteria to guarantee pathogen infection and transmission. To address this hypothesis, in this research a comparative proteomics approach was used to characterize host response to natural *M*. *bovis* infection using the wild boar TB model. The results identified host immune system proteins that are manipulated by mycobacteria for pathogen infection and transmission.

## Methods

### Ethics statement

All animal sampling was post-mortem. Wildlife samples were obtained from hunter-harvested individuals that were shot during the legal hunting season independently and prior to our research. According to EU and National legislation (2010/63/UE Directive and Spanish Royal Decree 53/2013) and to the University of Castilla–La Mancha guidelines no permission or consent is required for conducting the research reported here.

### Study site and sampling

Based on tooth eruption patterns [17], young (Age 6–24 months; N = 14) and adult (Age >24 months; N = 15) Eurasian wild boar were selected and included in the study. The animals were collected between hunting seasons 2009–2012 from Montes de Toledo, Spain in a region with 66±5% TB prevalence in wild boar [18]. The hunters provided the whole carcass less than 20 min after the animal died and the necropsy were performed on site [18]. Animals were subjected to detailed necropsy as described previously [14]. Samples of dissected mandibular lymph nodes were obtained by sagittal cross-section at half the length, and tissue fragments of approximately 2 cm^3^ were rapidly prepared and stored in liquid nitrogen for DNA, RNA and protein extraction. The remaining portion of the sample was used for culture and spoligotyping of mycobacteria (see below). After necropsy, young animals were classified as TB- (N = 5) or TB+ (N = 9) based on the absence/presence of TB-compatible lesions and negative/positive for mycobacterial culture. Adult animals were classified as TB- (N = 4), TB+ (N = 5) or TB++ (N = 6). The TB- animals were negative for mycobacteria culture and did not have TB-compatible lesions. The TB+ and TB++ animals were positive for mycobacteria culture and showed TB-compatible lesions localized in head organs (mandibular lymph nodes and/or oropharyngeal tonsils) referring to localized (potentially early) or controlled *M*. *bovis* infection or affecting multiple organs in the head and thorax that reflect disseminated TB, respectively (Table 1). Adult TB++ wild boar with disseminated disease showed extensive macroscopic lesions with poor fibrotic containment of the granulomas and ulceration into the lumina of airways. All animals positive for mycobacteria culture had infection with *M*. *bovis*. Mandibular lymph nodes were used in the study because these organs are involved in mycobacterial infection and TB [10, 14–16].

**Table 1:** Characterization of TB lesions in Young TB+, Adult TB+ and Adult TB++ wild boar included in the study.

Experimental group	Lesion localization (N)	Lesion score (Average ± SD)
Young TB+ (N = 9)	Mandibular lymph node (9)	7.2 ± 5.9
	Tonsil (2)	
	Bronchial (5)	
	Mediastinum (5)	
	Lung (2)	
	Spleen (1)	
	Mesentery (1)	
Adult TB+ (N = 5)	Mandibular lymph node (5)	2.4 ± 0.9
	Tonsil (1)	
Adult TB++ (N = 6)	Mandibular lymph node (6)	8.1 ± 4.2
	Bronchial (6)	
	Mediastinum (5)	
	Lung (2)	

Lesion scores were determined as previously described [19]. N, number of animals.

### Protein extraction and proteomics analysis

Proteins from mandibular lymph nodes were extracted using the AllPrep DNA/RNA/Protein Mini Kit (Qiagen, Inc. Valencia, CA, USA) according to manufacturer instructions. Precipitated proteins from individual samples of each group (TB- and TB+ young animals and TB-, TB+ and TB++ adult animals) were resuspended in 20mM Tris-HCl pH 7.5 with 4% SDS and protein concentration was determined using the BCA Protein Assay (Thermo Scientific, San Jose, CA, USA) using bovine serum albumin (BSA) as standard. Protein extracts (150 μg per sample) were on-gel concentrated by SDS-PAGE as previously described [20]. The unseparated protein bands were visualized by staining with GelCode Blue Stain Reagent (Thermo Scientific), excised, cut into 2 × 2 mm cubes and digested overnight at 37°C with 60 ng/μl sequencing grade trypsin (Promega, Madison, WI, USA) at 5:1 protein: trypsin (w/w) ratio in 50 mM ammonium bicarbonate, pH 8.8 containing 10% (v/v) acetonitrile [21]. The resulting tryptic peptides from each band were extracted by 30 min-incubation in 12 mM ammonium bicarbonate, pH 8.8. Trifluoroacetic acid was added to a final concentration of 1% and the peptides were finally desalted onto OMIX Pipette tips C18 (Agilent Technologies, Santa Clara, CA, USA), dried-down and stored at −20°C until mass spectrometry analysis.

The desalted protein digests was resuspended in 0.1% formic acid and analyzed by RP-LC-MS/MS using an Easy-nLC II system coupled to an ion trap LTQ mass spectrometer (Thermo Scientific). The peptides were concentrated (on-line) by reverse phase chromatography using a 0.1×20 mm C18 RP precolumn (Thermo Scientific), and then separated using a 0.075×100 mm C18 RP column (Thermo Scientific) operating at 0.3 ml/min. Peptides were eluted using a 180-min gradient from 5 to 40% solvent B (Solvent A: 0,1% formic acid in water, solvent B: 0,1% formic acid in acetonitrile). ESI ionization was done using a Fused-silica PicoTip Emitter ID 10 mm (New Objective, Woburn, MA, USA) interface. Peptides were detected in survey scans from 400 to 1600 amu (1 mscan), followed by fifteen data dependent MS/MS scans (Top 15), using an isolation width of 2 mass-to-charge ratio units, normalized collision energy of 35%, and dynamic exclusion applied during 30 sec periods.

The MS/MS raw files were searched against the Uniprot-*Sus scrofa* database (34,207 entries in November 2015) (http://www.uniprot.org) using the SEQUEST algorithm (Proteome Discoverer 1.4, Thermo Scientific). The following constraints were used for the searches: tryptic cleavage after Arg and Lys, up to two missed cleavage sites, and tolerances of 1 Da for precursor ions and 0.8 Da for MS/MS fragment ions and the searches were performed allowing optional Met oxidation and Cys carbamidomethylation. A false discovery rate (FDR) < 0.01 was considered as condition for successful peptide assignments and at least two peptide-spectrum matches (PSMs) per protein were the necessary condition for protein identification (S1 Table).

Gene ontology (GO) analysis for biological process (BP) was done by Blast2GO software (version 3.0; www.blast2go.com). Two biological replicates with 2–5 pooled mandibular lymph node samples each were used for analysis. Identified proteins were grouped according to BP GO. Within each BP, the average number of PSMs for each *S*. *scrofa* protein were added and normalized against the total number of PSMs and compared separately in young and adult animals between TB- and TB+ or between TB-, TB+ and TB++ samples, respectively by Chi2-test (p = 0.05). The average number of normalized PSMs for proteins in BPs with statistically significant differences between samples was then used to identify proteins with significant differences in representation within each BP by Chi2-test (p<0.05). The mass spectrometry proteomics data have been deposited at the ProteomeXchange Consortium (http://proteomecentral.proteomexchange.org) via the PRIDE partner repository with the dataset identifier PXD003251 and doi: 10.6019/PXD003251. S1 Table contains all *S*. *scrofa* proteins identified with FDR<0.01 and at least two PSMs per protein in at least one of the analyzed samples, BP annotation and data quantitation. Venn diagrams were constructed using Concept Draw PRO 10 (CS Odessa LLC, San Jose, CA, USA).

### Production of recombinant proteins and rabbit antibodies

Recombinant Calcium binding protein A9 (S100A9; Uniprot accession number C3S7K6), Lactotransferrin (LTF; Q6YT39) and Peptidoglycan-recognition protein (PGLYRP1; F1RM24) were produced in *Escherichia coli* using pET101/D-TOPO expression system (Invitrogen-Life Technologies Inc, Grand Island, NY, USA). Coding regions were amplified by RT-PCR using total RNA extracted from 3D4/31 cell line (Pig macrophage) samples and sequence-specific oligonucleotide primers (S2 Table) and cloned into the pET101/D-TOPO expression vector (Invitrogen-Life Technologies Inc, Grand Island, NY, USA). Recombinant proteins were purified by Ni affinity chromatography to >95% purity and used to produce antibodies in rabbits as previously described [22]. The specificity of the antibodies was characterized by Western blot as described below but using 10 μg of recombinant proteins.

### Western blot analysis

For Western blot analysis, 15 μg of total proteins from individual wild boar mandibular lymph nodes (young TB-, N = 5; young TB+, N = 9; adult TB-, N = 4; adult TB+, N = 5; adult TB++, N = 5) were loaded onto a 12% SDS-polyacrylamide gel (Life Science, Hercules, CA, USA) and transferred to a nitrocellulose membrane during 1 h at 12 V in a Mini-Genie Electroblotter semi-dry transfer unit (Idea Scientific, Corvallis, OR, USA). The membrane was blocked with 5% skim milk for 1 h at room temperature, washed three times in TBS and probed with rabbit antibodies. Serum from rabbits immunized with recombinant proteins was diluted 1:500 in 3% BSA in TBS and the membrane was incubated with the diluted sera for 1 h at room temperature, and washed three times with TBS. Rabbit antibodies against the recombinant 40S ribosomal protein S14 (RPS14; Q29303) (Sigma-Aldrich Co, St. Louis, MO, USA) were used as control for normalization in Western blot analysis. The membrane was then incubated with an anti-rabbit horseradish peroxidase (HRP) conjugate (Sigma-Aldrich Co, St. Louis, MO, USA) diluted 1:1000 in TBS. The membrane was washed three times with TBS and finally developed with TMB stabilized substrate for HRP (Promega) for 20 min. The intensity of protein bands corresponding to test and control proteins including those with lower or higher molecular weight than the recombinant protein that likely correspond to degradation and polymerization products, respectively were determined in the Western blot membrane by densitometric analysis using ImageJ 1.44p (National institute of Health, USA). The intensity of test protein bands was normalized against the intensity of the control band and analyzed by a multivariate comparison between the groups using the one-way ANOVA test followed by one-tailed Student’s t-test with Bonferroni correction for samples with unequal variance (p = 0.05).

### Determination of hemoglobin protein levels

Hemoglobin protein levels were determined by ELISA (Cloud-Clone Corp., Houston, TX, USA) in serum from individual wild boar (young TB-, N = 4; young TB+, N = 7; adult TB-, N = 4; adult TB+, N = 5; adult TB++, N = 6). Optical density values were converted to g/dl Hemoglobin using the ELISA standard curve and compared between groups by one-tailed Student’s t-test for samples with unequal variance (p = 0.05).

### Spoligotyping of mycobacteria

Pools of mandibular lymph node samples were submitted to culture and spoligotyping of mycobacteria as previously described [23, 24]. The frequency of different spoligotypes in each group was compared between groups by ANOVA F-test (p = 0.05).

### RNA extraction and quantitative real-time RT-PCR

Total RNA was isolated from individual wild boar mandibular lymph nodes tissue samples using the AllPrep DNA/RNA/Protein Mini Kit (Qiagen, Inc. Valencia, CA, USA) according to manufacturer’s instructions. Individual RNA samples of young TB-, TB+ and adult TB-, TB+, TB++ wild boar mandibular lymph nodes (young TB-, N = 5; young TB+, N = 9; adult TB-, N = 4; adult TB+, N = 5; adult TB++, N = 5) were used for real-time RT-PCR analysis. Primers were synthesized based on the sequences determined for *S*. *scrofa C3* [12], *MUT* [12], *S100A9*, *LTF*, and *PGLYRP1* genes (S2 Table). Real-time RT-PCR was performed using the QuantiTec SYBR Green RT-PCR kit and a Rotor Gene Q thermocycler (Qiagen, Inc. Valencia, CA, USA) following manufacturer’s recommendations. Amplification efficiencies were normalized against *S*. *scrofa cyclophilin* and expressed as transcript copy numbers in arbitrary units [14–16]. Pair comparisons between mRNA expression levels were done by a multivariate comparison between the groups using the one-way ANOVA test followed by one-tailed Student’s t-test with Bonferroni correction for samples with unequal variance (p = 0.05).

### Analysis of experimentally infected wild boar

For controlled experimental infection with *M*. *bovis*, wild boar were selected from the control group in the vaccine trial previously reported [8]. Selected infected wild boar were divided into two groups after necropsy. TB+ animals (N = 2) had a 6–12 lesion score with TB lesions in the mandibular (N = 2) and tracheobronchial (N = 1) lymph nodes [8]. TB++ animals (N = 3) had a 16–38 lesion score with TB lesions in tonsils (N = 1), mandibular (N = 3), retropharyngeal (N = 3), tracheobronchial (N = 3) lymph nodes and lungs (N = 2) [8]. All animals were positive for *M*. *bovis* cultures [8]. Proteins from tonsils of TB+ and TB++ wild boar were extracted, on-gel concentrated, trypsin digested and analyzed by RP-LC-MS/MS following the same procedures described above for field-collected samples. The MS/MS raw files were searched against the Uniprot-*Sus scrofa* database (34,207 entries in November 2015) (http://www.uniprot.org) using the SEQUEST algorithm (Proteome Discoverer 1.4, Thermo Scientific) with the same constraints described above. A FDR < 0.01 was considered as condition for successful peptide assignments and at least two PSMs per protein were the necessary condition for protein identification (S3 Table). For targeted proteomics, the MS/MS raw files were searched against a database composed of the six differentially represented immune system proteins in naturally infected wild boar (C3S7K6, F1RRP1, Q6YT39, Q8SPA3, I3LNT1 and F1RM24) plus control Actin (P68137) using the SEQUEST algorithm (Proteome Discoverer 1.4, Thermo Scientific) with the same constraints described above. A FDR < 0.05 was considered as condition for successful peptide assignments and subsequent protein identification (S3 Table). Two (TB+ animals) or three (TB++ animals) biological replicates were used for analysis. The average number of PSMs for each *S*. *scrofa* protein were added and normalized against the total number of PSMs and compared between TB+ and TB++ samples by Chi2-test (p = 0.05). S3 Table contains all *S*. *scrofa* proteins identified with FDR<0.01 and at least two PSMs per protein in at least one of the samples and the *S*. *scrofa* proteins analyzed with FDR<0.05 in targeted proteomics.

## Results

### Comparative proteomics identifies host immune system proteins affected by infection with *M*. *bovis*

**Fig 1:**
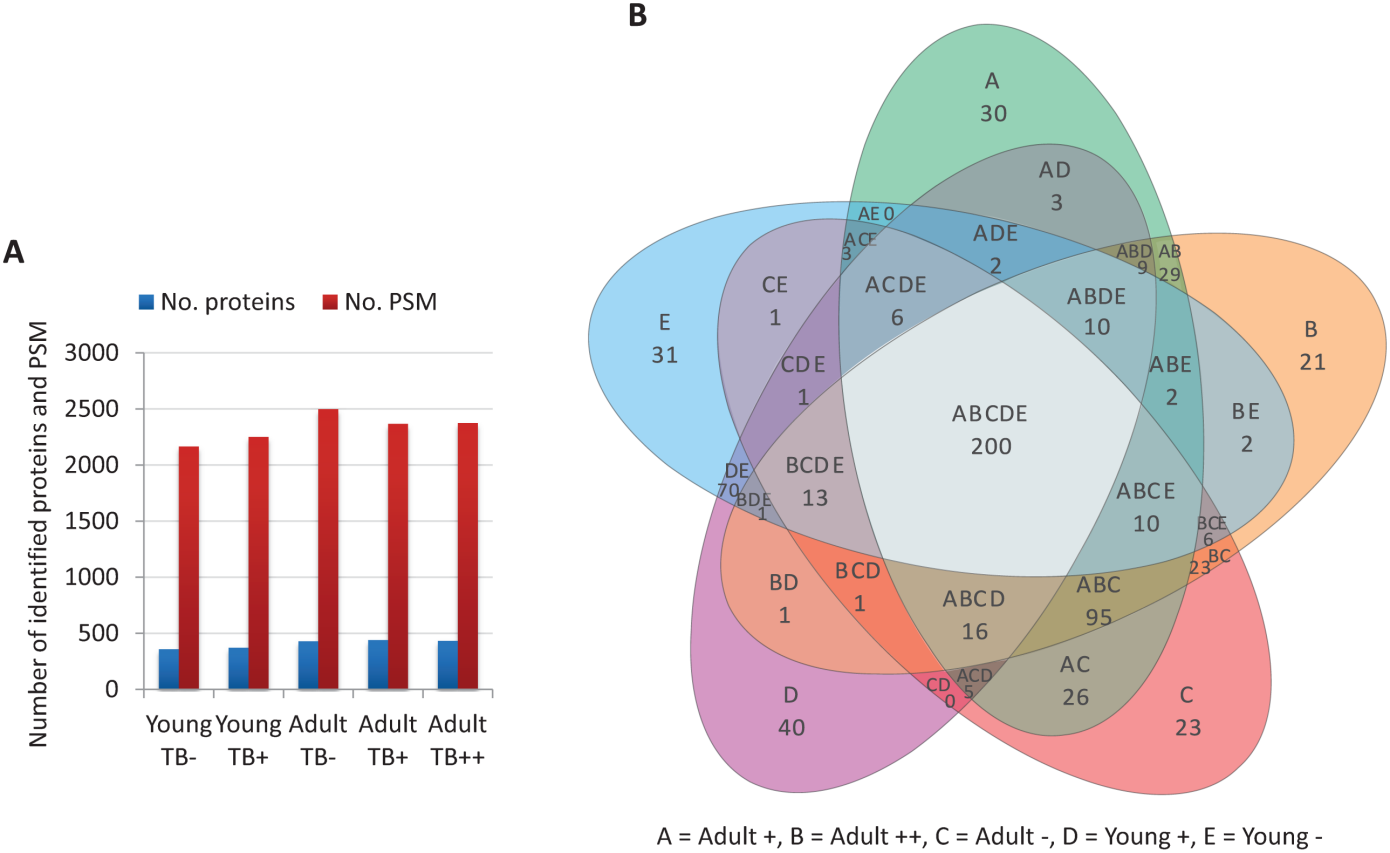
Proteomics results in wild boar mandibular lymph nodes. (A) Proteins were identified with FDR<0.01 and at least two peptides per protein in at least one of the analyzed samples and quantitated using PSM. (B) Venn diagram showing protein distribution between different experimental groups included in the study.

After proteomics analysis of wild boar mandibular lymph nodes, a total of 428 and 532 proteins were identified in young and adult animals, respectively (S1 Table). The number of identified proteins and PSM with which these proteins were identified was similar between experimental groups (young TB-, young TB+, adult TB-, adult TB+, adult TB++) ranging from 358 to 439 proteins and 2165–2497 PSM (Fig 1A). As expected, the same proteins were identified in several experimental groups with 200 proteins found in all groups (Fig 1B).

**Fig 2:**
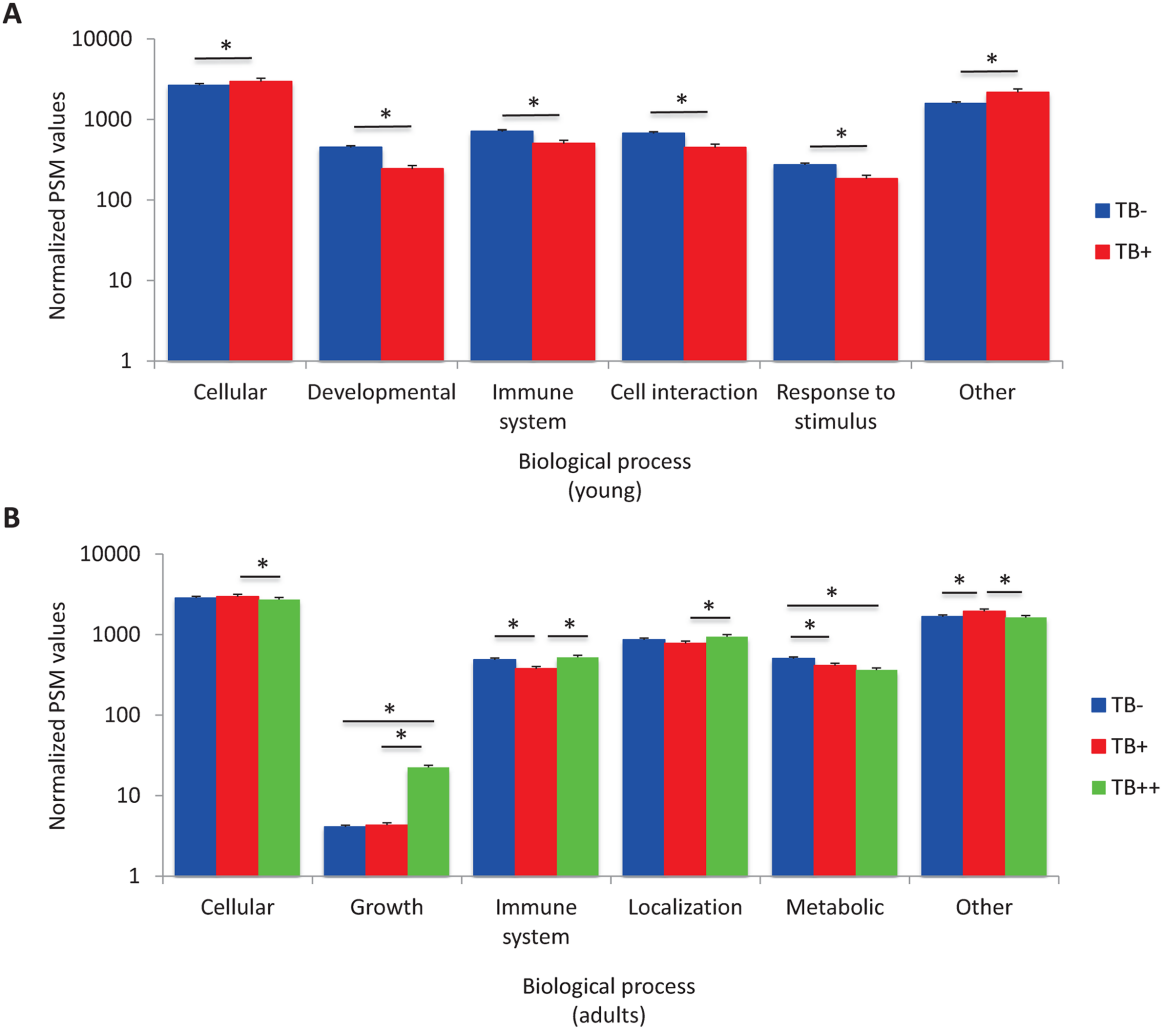
Comparative proteomics of mandibular lymph nodes from *M*. *bovis*-infected and uninfected wild boar. Two biological replicates with 2–5 pooled mandibular lymph node samples each were used for comparative proteomics analysis. Identified proteins were grouped according to BP GO using Blast2GO. Within each BP, the average number of PSMs for each *S*. *scrofa* protein were added and normalized against the total number of PSMs and compared between infected and uninfected animals by Chi2-test (*p<0.05). (A) Comparative analysis between uninfected (TB-) and infected (TB+) young animals. (B) Comparative analysis between uninfected (TB-), infected with TB lesions localized in the head (TB+) and infected with TB lesions affecting multiple organs (TB++) adult animals.

The GO analysis showed that the most represented BPs were cellular, immune system, cell interaction, developmental and response to stimulus processes in young wild boar (Fig 2A), while cellular, immune system, localization, metabolic and growth processes were the BPs with most represented proteins in adult animals (Fig 2B). Significant differences were observed in the most represented BPs between uninfected and *M*. *bovis*-infected young and adult wild boar (Fig 2A and 2B) or between TB+ and TB++ adult animals (Fig 2B). Two BPs, cellular and immune system were represented in both young and adult animals (Fig 2A and 2B).

**Fig 3:**
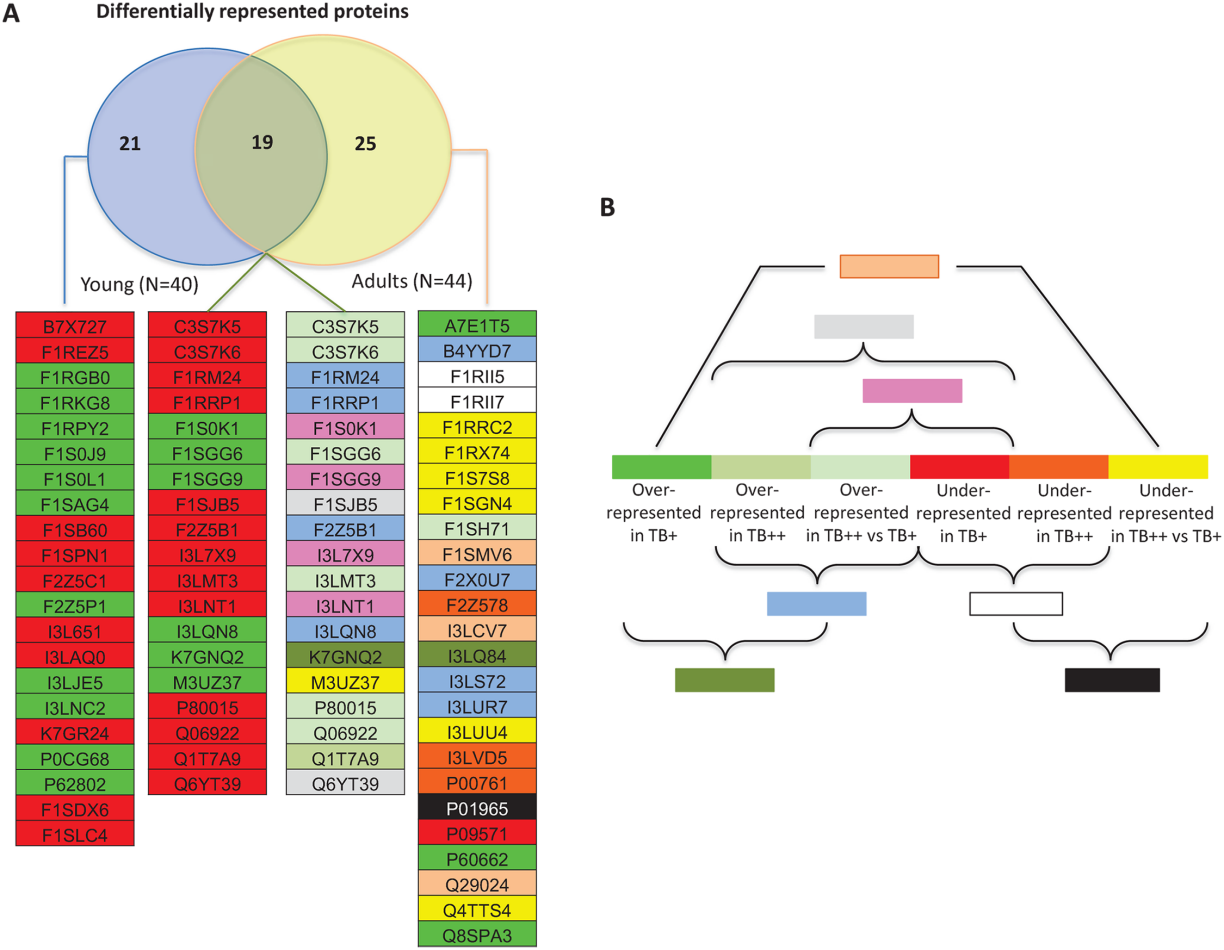
Differentially represented proteins between *M*. *bovis*-infected and uninfected wild boar. The average number of normalized PSMs for proteins in BPs with statistically significant differences between samples was used to identify proteins with significant differences in representation within each BP by Chi2-test (p<0.05). (A) Venn diagram and annotation of the differentially represented proteins in young and adult wild boar. Uniprot accession numbers are shown. (B) Color code for over-represented or under-represented proteins when comparing infected (TB+) and uninfected (TB-) young animals or uninfected (TB-), infected with TB lesions localized in the head (TB+) and infected with TB lesions affecting multiple organs (TB++) adult animals.

Protein quantitative analysis within each of the most represented BPs resulted in 40 and 44 differentially represented proteins in young and adults, respectively (Fig 3A). Of them, 19 proteins were differentially represented in both young and adult wild boar (Fig 3A). Differentially represented proteins were over-represented or under-represented in TB+ young animals when compared to TB- uninfected controls (Fig 3A and 3B). In adult animals, differentially represented proteins showed a complex pattern when comparing the different groups (Fig 3A and 3B).

**Fig 4:**
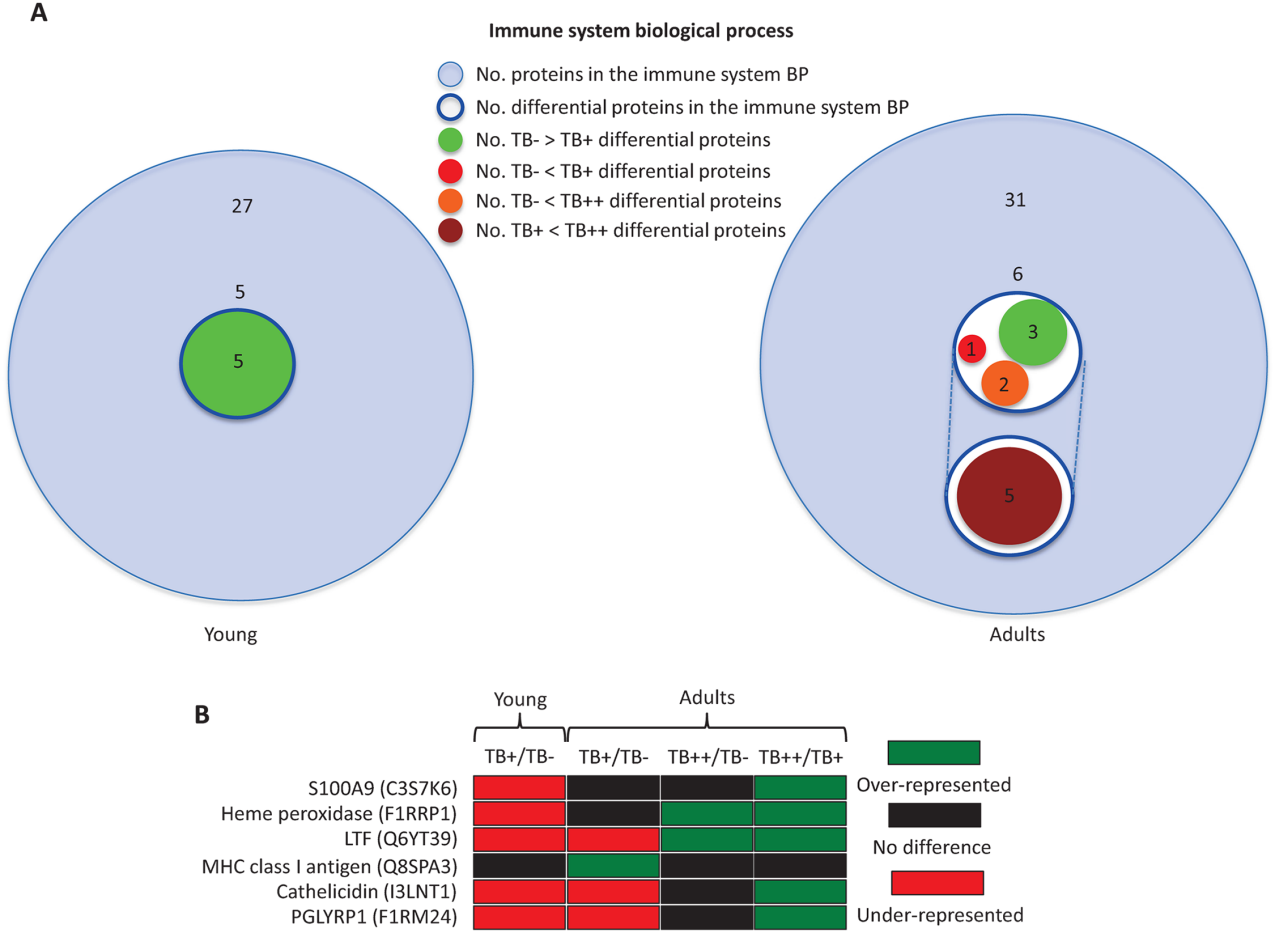
Characterization of immune system proteins. The immune system BP was the only with significant differences between infected and uninfected animals in both young and adults and was selected for further analysis. (A) Venn diagram of immune system proteins in young and adult animals, showing the number of differentially under-represented and over-represented proteins when comparing infected (TB+) and uninfected (TB-) young animals or uninfected (TB-), infected with TB lesions localized in the head (TB+) and infected with TB lesions affecting multiple organs (TB++) adult animals. (B) Representation profile of immune system differentially represented proteins in young and adult animals.

Of the BPs represented in young and adult wild boar, only the immune system BP was significantly different between infected and uninfected animals in both age groups (Fig 2A and 2B). Immune system proteins play an important role in host response to mycobacteria and other infectious microorganisms and were therefore selected for further analysis. A total of 27 and 31 proteins were included into the immune system BP in young and adult animals, respectively (Fig 4A). Of them, 5 and 6 proteins were differentially represented in young and adult animals, respectively (Fig 4A). In young animals, all 5 immune system proteins S100A9 (C3S7K6), uncharacterized Heme peroxidase (F1RRP1), LTF (Q6YT39), uncharacterized Cathelicidin (I3LNT1) and PGLYRP1 (F1RM24) were under-represented in TB+ animals when compared to uninfected TB- controls (Fig 4A and 4B). In adults, 3 proteins (LTF, Cathelicidin, PGLYRP1) had the same representation than in young animals while the other 3 proteins were over-represented in infected TB+ (MHC class I antigen, MHCI; Q8SPA3) or TB++ (Heme peroxidase, LTF) animals when compared to uninfected TB- controls (Fig 4A and 4B). Additionally, 5 of the differentially represented proteins in adults (S100A9, Heme peroxidase, LTF, Cathelicidin, PGLYRP1) were over-represented in TB++ when compared to TB+ animals (Fig 4A and 4B).

**Fig 5:**
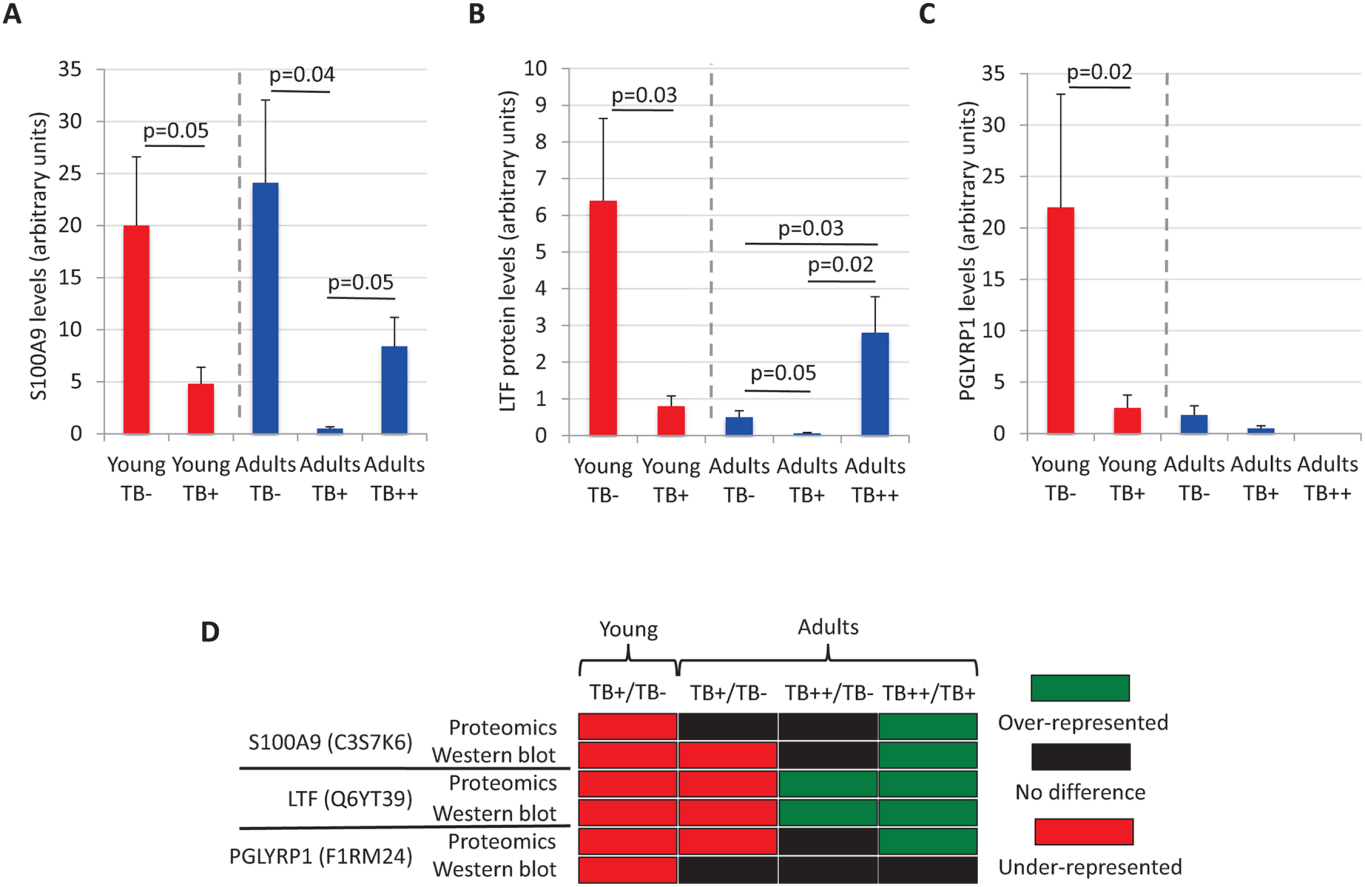
Validation of proteomics results by Western blot analysis. To validate proteomics results, selected differentially represented immune system proteins were produced in *E*. *coli* and used to generate antibodies in rabbits for Western blot analysis of individual wild boar mandibular lymph node protein samples (young TB-, N = 5; young TB+, N = 9; adult TB-, N = 4; adult TB+, N = 5; adult TB++, N = 5). The intensity of protein bands corresponding to test and control RPS14 proteins was determined by densitometric analysis. The intensity of test protein bands was normalized against the intensity of the control band, represented as average + S.D. and compared between groups in adult or young wild boars by a multivariate comparison between the groups using the one-way ANOVA test followed by one-tailed Student’s t-test with Bonferroni correction for samples with unequal variance (p = 0.05). (A) Normalized S100A9 protein levels. (B) Normalized LTF protein levels. (C) Normalized PGLYRP1 protein levels. (D) Comparative analysis of proteomics and Western blot results for differentially represented immune system proteins S100A9, LTF and PGLYRP1.

To validate proteomics results, differentially represented immune system proteins S100A9, LTF and PGLYRP1 were produced in *E*. *coli* (S1A Fig) and used to generate rabbit antibodies specific for recombinant *S*. *scrofa* proteins (S1B Fig). The Western blot analysis of individual wild boar mandibular lymph node protein samples (S1C–S1E Fig) showed a good correlation with proteomics results and validated proteomics results for these proteins (Fig 5A–5D).

To provide additional support for the results obtained in naturally infected animals, wild boar experimentally infected with *M*. *bovis* under controlled conditions [8] were used to characterize the levels of differentially represented immune system proteins C3S7K6, F1RRP1, Q6YT39, Q8SPA3, I3LNT1 and F1RM24 by targeted proteomics (S3 Table). Mandibular lymph nodes were not available for analysis. Therefore, tonsils that are also involved in mycobacterial infection and TB [14–16, 19] were used for analysis. The results were similar between TB+ and TB++ experimentally infected and naturally infected animals for most of the differentially represented immune system proteins (S2 Fig).

### Hemoglobin protein levels decrease with *M*. *bovis* infection in adult wild boar

**Fig 6:**
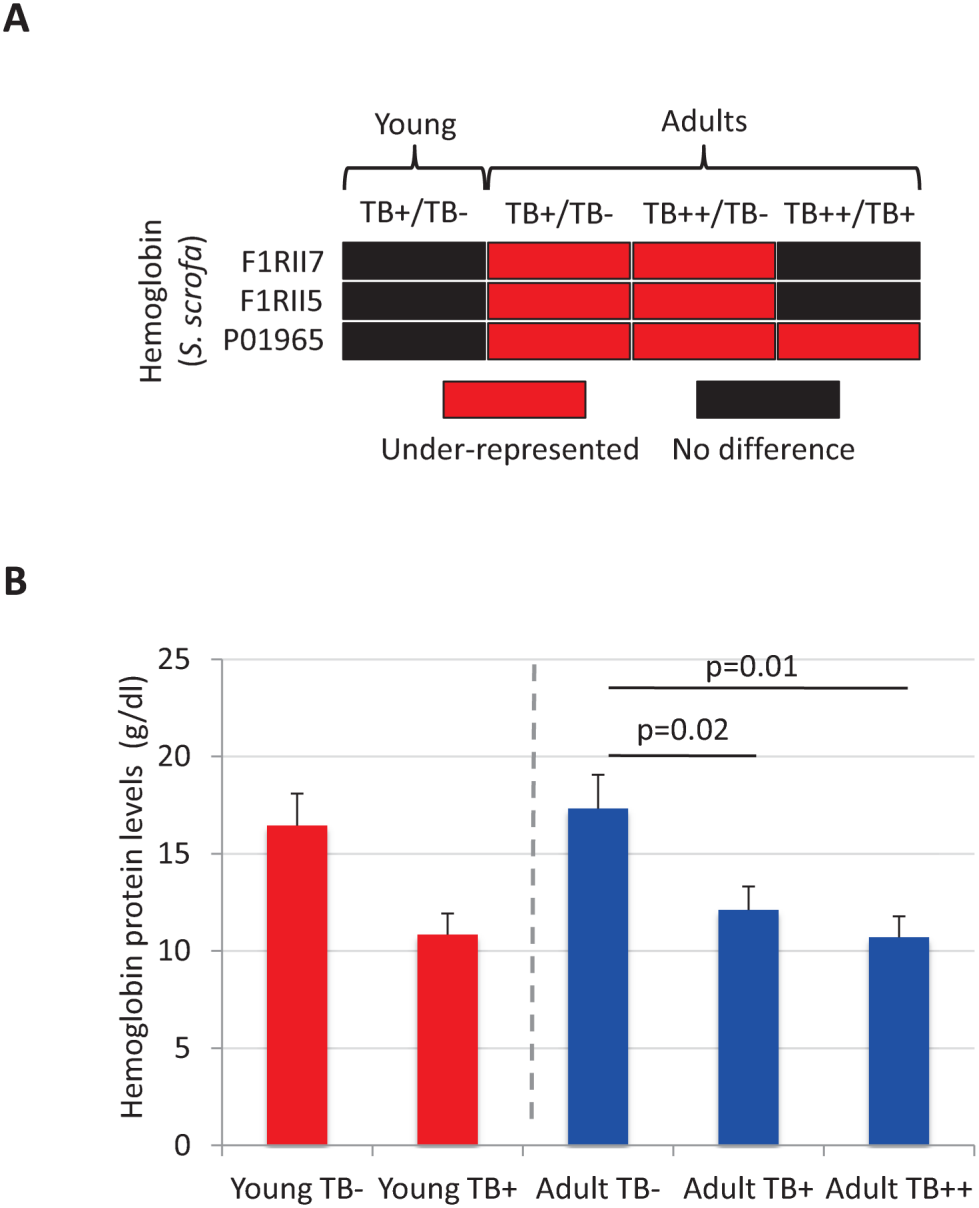
Characterization of Hemoglobin protein levels. (A) Representation of proteomics results showing that *S*. *scrofa* Hemoglobin proteins F1RII7, F1RII5 and P01965 were under-represented in *M*. *bovis*-infected adult wild boar when compared to uninfected animals. (B) Hemoglobin protein levels were determined by ELISA in serum from individual wild boar (young TB-, N = 4; young TB+, N = 7; adult TB-, N = 4; adult TB+, N = 5; adult TB++, N = 6). Optical density values were converted to g/dl Hemoglobin using the ELISA standard curve, represented as average + S.D. and compared between groups by a multivariate comparison between the groups using the one-way ANOVA test followed by one-tailed Student’s t-test with Bonferroni correction for samples with unequal variance (p = 0.05).

Proteomics results showed that Hemoglobin proteins were under-represented in *M*. *bovis*-infected adult wild boar when compared to uninfected animals (Fig 6A). These results were validated by ELISA in individual adult wild boar serum (Fig 6B). In young animals, a tendency was observed towards lower Hemoglobin levels in infected animals, but results were not statistically significant due to high individual variation in protein levels (Fig 6B).

### Differentially represented immune system proteins may be regulated at the transcriptional and post-transcriptional levels

**Fig 7:**
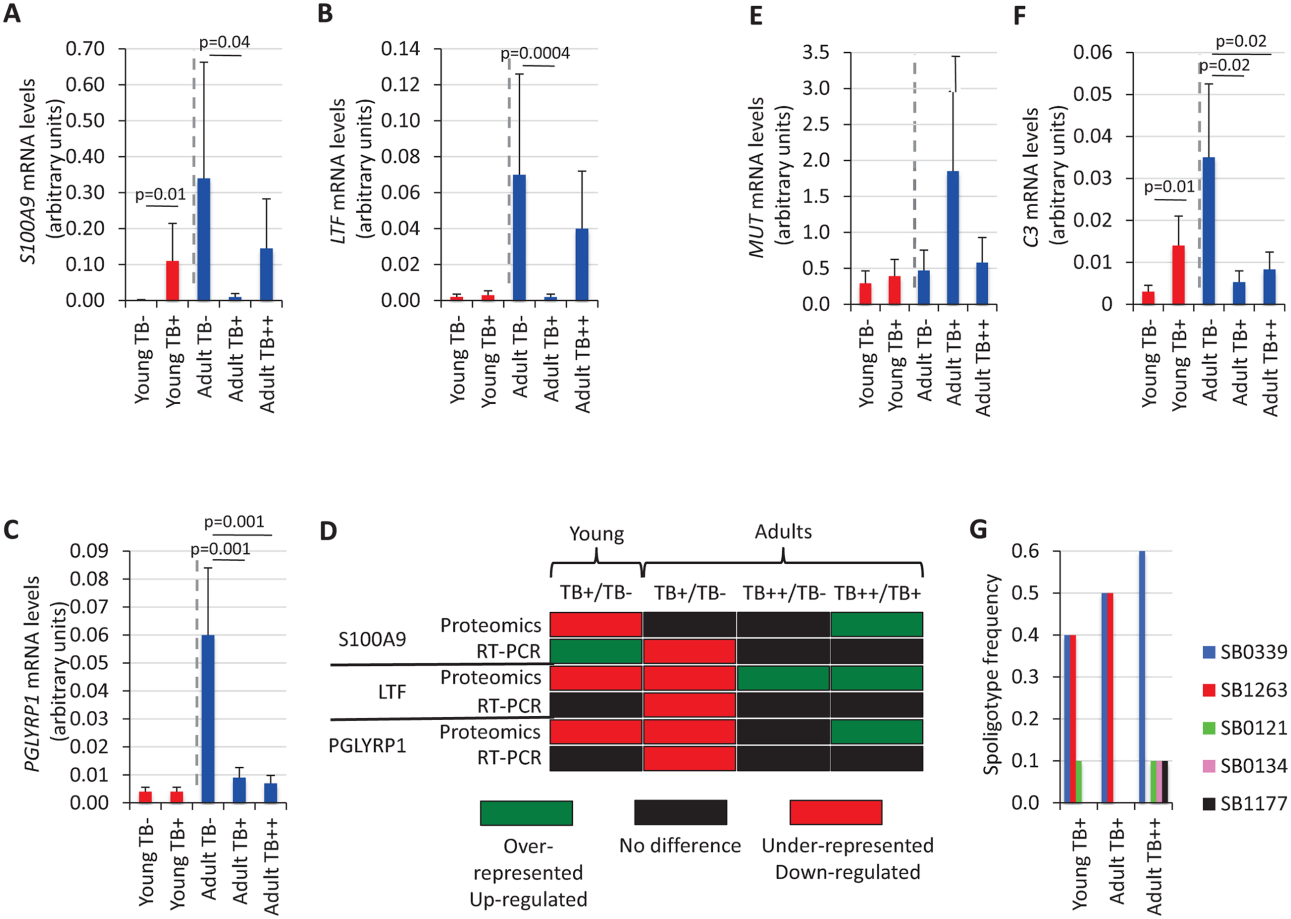
Characterization of transcriptional response for selected genes and *M*. *bovis* spoligotype composition in wild boar. (A) Normalized *S100A9* mRNA levels. (B) Normalized *LTF* mRNA levels. (C) Normalized *PGLYRP1* mRNA levels. (D) Comparative analysis of proteomics and RT-PCR results for differentially represented immune system proteins S100A9, LTF and PGLYRP1. (E) Normalized *MUT* mRNA levels. (F) Normalized *C3* mRNA levels. Individual RNA samples of young and adult wild boar mandibular lymph nodes (young TB-, N = 5; young TB+, N = 9; adult TB-, N = 4; adult TB+, N = 5; adult TB++, N = 5) were used for real-time RT-PCR analysis. Amplification efficiencies were normalized against *S*. *scrofa cyclophilin*, expressed as transcript copy numbers in arbitrary units and represented as average + S.D. Pair comparisons between mRNA expression levels were done by one-tailed Student’s t-test for samples with unequal variance (p = 0.05). (G) *M*. *bovis* spoligotype frequency identified in wild boar included in the study.

To further characterize the response mediated by immune system proteins differentially represented in response to *M*. *bovis* infection, a transcriptional profile was obtained in mandibular lymph nodes for genes coding for S100A9 (Fig 7A), LTF (Fig 7B) and PGLYRP1 (Fig 7C). The results did not show correlation between protein and mRNA levels in young animals or when comparing adult TB++ and TB+ animals (Fig 7D). However, in adult wild boar a 67% (2/3) correlation was obtained when comparing mRNA and protein levels between TB- and TB+ or TB++ animals (Fig 7D).

### Host and pathogen genetic factors may impact on *M*. *bovis* infection and disease in wild boar

The impact of host and pathogen genetic factors on *M*. *bovis* infection and disease has been documented in the wild boar TB model [10, 14–16, 24]. The expression of *MUT* and *C3* was characterized in young and adult wild boar (Fig 7E and 7F). The results did not support a role for *MUT* in wild boar infection by *M*. *bovis* in this population (Fig 7E). However, *C3* mRNA levels were higher in young TB+ animals but lower in infected TB+ and TB++ adult animals when compared to uninfected TB- controls (Fig 7F), suggesting a role for *C3* in host response to *M*. *bovis* infection.

Five different *M*. *bovis* spoligotypes (SB0339, SB1263, SB0121, SB0134, SB1177) were identified in wild boar included in the study (Fig 7G). Although some spoligotypes were not present in all groups, the frequency of different spoligotypes was not statistically different between groups (p = 0.99) (Fig 7G). Adult animals had higher spoligotype diversity. The SB0339 spoligotype was isolated with the highest frequency (0.4–0.6) from all groups (Fig 7G), while spoligotypes SB0134 and SB01177 were identified with low frequency (0.1) only in adult TB++ animals (Fig 7G).

## Discussion

This study was based on wild boar naturally exposed to *M*. *bovis* as a model to characterize host response to mycobacterial infection with emphasis on immune system proteins. Natural infections reflect better the conditions found in the field and therefore increase the relevance of the results for the characterization of mycobacterial infection under natural conditions [14]. However, as previously discussed [14], this approach did have some disadvantages such as the presence of mixed infections in the animals. Nevertheless, it was possible to minimize the effect of these mixed infections on differential gene expression and protein representation analyses because they were equally present in all the TB- and TB+/TB++ animals. Additionally, results obtained in TB+ and TB++ animals experimentally infected under controlled conditions corroborated the findings in naturally infected animals for most of the differentially represented immune system proteins. Mandibular lymph nodes were used because these organs are involved in mycobacterial infection and TB [14–16, 19]. Our hypothesis is that immune system proteins under-represented in infected animals when compared to uninfected controls are affected by mycobacteria to facilitate pathogen infection and transmission. To address this hypothesis, in this research a comparative proteomics approach was used to compare host response between uninfected (TB-) and *M*. *bovis*-infected young (TB+) and adult animals with different infection status [TB lesions localized in the head (TB+) referring to localized (potentially early) or controlled *M*. *bovis* infection or affecting multiple organs (TB++) that reflect disseminated TB]. The results suggested an effect of mycobacterial infection on proteins affecting different BP and MF of which immune system BP showed significant differences between uninfected and infected wild boar in both young and adult animals.

The analysis was then focused on the immune system proteins that play an important role in host response to mycobacteria [9, 14–16]. Five and six immune response proteins were differentially represented in young and adult animals, respectively. Proteins S100A9, Heme peroxidase, LTF, Cathelicidin and PGLYRP1 were under-represented in TB+ animals when compared to uninfected TB- controls but protein levels increased as infection progressed in TB++ animals when compared to TB- and/or TB+ adult wild boar. MHCI was the only protein over-represented in TB+ adult wild boar when compared to uninfected TB- controls. For selected differentially represented immune system proteins, proteomics results using pooled samples were validated by Western blot analysis in individual wild boar mandibular lymph nodes therefore providing support for the results reported here.

The differentially represented immune system proteins were all related to host immune response to mycobacterial infection with relevant implication in pathogen infection, multiplication and transmission.

### Calcium binding protein A9 (S100A9)

The protein S100A9 is produced by neutrophils and has been suggested to be involved in the positive regulation of intrinsic apoptotic signaling pathway, innate immune response and autophagy among other processes [25], all mechanisms involved in host immune response to mycobacterial infection [26]. Therefore, reducing S100A9 protein levels may be a mechanism used by *M*. *bovis* to evade host immune response and establish infection in young wild boar. However, S100A9 also mediates neutrophilic inflammation and lung pathology during active TB [27]. The over-representation of S100A9 in TB++ adult wild boar may be a host response to limit pathogen multiplication but it is also associated with active TB resulting in increased transmission of mycobacteria.

### Heme peroxidase

Heme is an important prosthetic group in hemoglobins, peroxidases, catalases, hydroxylases, and cytochromes required for various processes such as DNA transcription, RNA translation, protein stability, cell differentiation and immunity [28]. Heme peroxidase such as Eosinophil peroxidase also shows inhibitory activity against mycobacteria by inducing bacterial fragmentation and lysis [29]. Furthermore, most bacterial pathogens including mycobacteria require heme and iron for full virulence and have developed systems for heme acquisition [28, 30]. Therefore, the under representation of Heme peroxidase in infected TB+ wild boar when compared to uninfected TB- animals may be induced by *M*. *bovis* to evade host immune response and establish infection in young animals. As infection proceeds in adult wild boar to affect several organs in TB++ animals, Heme peroxidase protein levels are higher than in TB+ animals which may represents a mechanism for the host to inhibit pathogen multiplication. However, mycobacteria may benefit from this response in adult TB++ wild boar by acquiring Heme to increase virulence and favor transmission.

### Lactotransferrin (LTF)

Mycobacteria of the MTBC grow within macrophages to establish infection in the host [11]. Iron (Fe) acquisition is critical for mycobacterial growth and bacteria acquire Fe bound to citrate, Transferrin and LTF and from macrophage cytoplasm [31]. Furthermore, host immune response to mycobacteria infection partly depends on iron regulation by the host through the tight control of iron-storage proteins [32,33]. Consequently, LTF has been proposed as an adjuvant for the BCG vaccine to increase its efficacy [34]. Considering the critical role that LTF plays during mycobacterial infection, protein under-representation in TB+ young and adult animals when compared to uninfected TB- controls could reflect a mechanism of host immune response to infection by reducing Fe source to mycobacteria. However, the LTF protein levels increased in TB++ adult animals suggesting a mechanism by which mycobacteria manipulate host immune response during infection progression to increase Fe availability resulting in higher bacterial growth and transmission.

### Cathelicidin

It has long been recognized that many people and animals exposed to MTBC do not subsequently show any evidence of infection probably due to innate, non-specific inflammatory responses that control infection or reduce the infection load, therefore modulating the subsequent host acquired immune response [35]. Cathelicidin is one of the antimycobacterial peptides delivered to phagosomes containing mycobacteria through fusion with lysosomes resulting in macrophage autophagy killing intracellular mycobacteria [35]. Therefore, reduction in the production of antimycobacterial peptides such as Cathelicidin increases susceptibility to TB [35]. As with other immune system proteins identified here as differentially represented in mandibular lymph nodes of naturally infected wild boar when compared to uninfected controls, Cathelicidin was under-represented in infected TB+ young and adult wild boar, probably reflecting a mechanism by which mycobacteria manipulate host innate immune response to facilitate infection and multiplication. However, Cathelicidin protein levels increased in TB++ adult animals suggesting a host mechanism to limit bacterial multiplication as infection progresses to increase host survival. Mycobacteria may benefit from this response in TB++ adult animals by increasing the probability of transmission to susceptible hosts.

### Peptidoglycan-recognition protein (PGLYRP1)

Peptidoglycan recognition proteins are part of the innate immune system that bind to bacterial cell wall molecules such as lipopolysaccharide, lipoteichoic acid, peptidoglycan and fatty acids such as mycobacterial mycolic acid [36]. The PGLYRP1 protein showed a profile similar to Cathelicidin in response to *M*. *bovis* infection in wild boar, again suggesting a mechanism by which mycobacteria manipulate host innate immune response to facilitate infection and multiplication but host response increases protein levels to limit bacterial multiplication as infection progresses to increase host survival. As discussed above, mycobacteria may benefit from this response in TB++ adult animals by increasing the probability of transmission to susceptible hosts.

### MHC class I antigen (MHCI)

MHCI was the only protein over-represented in TB+ adult wild boar when compared to uninfected TB- controls with no significant differences between other groups. According to the protein annotation, this MHCI antigen probably belongs to the classical MHC class Ia which functions by presenting peptide antigens to pathogen-specific cytotoxic T cells [37]. Therefore, the over-representation of MHCI in TB+ wild boar probably reflects host immune response to *M*. *bovis* infection. However, the T cell epitopes of MTBC including *M*. *bovis* are hyperconserved in different strains consistent with strong purifying selection acting on these epitopes [24, 38]. Consequently, MTBC might benefit from recognition by T cells because this essential response for host survival may be necessary for mycobacteria to establish latent infection [38].

Protein levels and the expression of coding genes for differentially represented immune system proteins were characterized by Western blot and real-time RT-PCR in individual wild boar mandibular lymph node protein and RNA samples, respectively. Western blot analysis validated the proteomics results. Additionally, Hemoglobin protein levels that were under-represented in *M*. *bovis*-infected adult wild boar when compared to uninfected animals were also validated by ELISA in individual wild boar serum samples. These results suggested the presence of regulatory mechanisms acting at both transcriptional and post-transcriptional levels depending on the age and infection status of the animals. In young infected animals, regulation was probably at the post-transcriptional level while in adult TB+ and TB++ animals the presence of transcriptional mechanisms was more evident. However, the comparison between TB++ and TB+ adult animals also suggested regulation at the post-transcriptional level to explain differences between mRNA and protein levels. Nevertheless, the discrepancy between mRNA and protein levels could also be explained by delay between mRNA and protein accumulation, which requires sampling at different time points. Immune response is regulated at both transcriptional and post-transcriptional levels [39] and the regulatory mechanisms that occur at the level of mRNA splicing, mRNA polyadenylation, mRNA stability and protein translation have instrumental roles in controlling both the magnitude and duration of the immune response [40]. Therefore, it was not surprising to find that both transcriptional and post-transcriptional mechanisms probably operated to regulate the levels of the immune system proteins identified as differentially represented in wild boar mandibular lymph node response to mycobacterial infection.

With the approach used in this research we were able to characterize the dynamics of wild boar immune response at the host-mycobacteria interface. However, other factors such as contact probability between hosts and mycobacteria and genetic factors of both hosts and pathogens could also affect infection prevalence and disease progression [9, 14–16, 24]. The contact between hosts and mycobacteria was very probable in adult animals due to the high (66%) *M*. *bovis* prevalence in wild boar in this region. Consequently, as shown in previous studies [14], uninfected adult animals were probably resistant to *M*. *bovis*. However, uninfected young animals could have been naïve to *M*. *bovis* infection.

To address the impact of host genetic factors on infection and disease progression, the expression of genes coding for C3 and MUT was characterized as possible correlates with host resistance to natural mycobacterial infection [9, 14–16]. The results did not support a role for *MUT* in susceptibility to *M*. *bovis* in these animals [16]. However, the results of *C3* expression supported a role for this molecule in the outcome of *M*. *bovis* infection. As reported in previous studies [8, 9], *C3* mRNA levels increased with *M*. *bovis* infection in young animals as a host response to limit mycobacterial infection. However, in adult wild boar higher *C3* levels correlated with protection against *M*. *bovis* infection, providing additional support for the central role of this molecule in the protective response against mycobacterial infection [8, 9, 14, 15]. Hemoglobin protein levels that are associated with TB-induced anemia [41] were lower in infected TB+ and TB++ adult animals when compared to uninfected controls, indicating anemia in infected wild boar, but without differences as infection progressed between TB+ and TB++ animals. The tendency in Hemoglobin protein levels in young wild boar also suggested anemia in infected animals.

Finally, the prevalence of *M*. *bovis* spoligotypes in infected animals was used to characterize pathogen genetic diversity in these animals and their possible impact on infection and disease [24]. Although the frequency of the 5 spoligotypes identified was not different between groups, adult animals had a greater spoligotype diversity probably reflecting a longer exposure to mycobacteria. Two of the spoligotypes, SB0339 and SB0134 has been phenotypically and genetically correlated with high distribution (isolation frequency) and low and high TB lesion score in wild boar, respectively [24]. The SB0339 spoligotype was isolated from all groups with high frequency but the SB0134 sopligotype was identified only in adult TB++ animals. These results are in agreement with previous reports [24] and suggest that mycobacteria-derived genetic factors may impact on *M*. *bovis* infection and disease in the study site.

**Fig 8:**
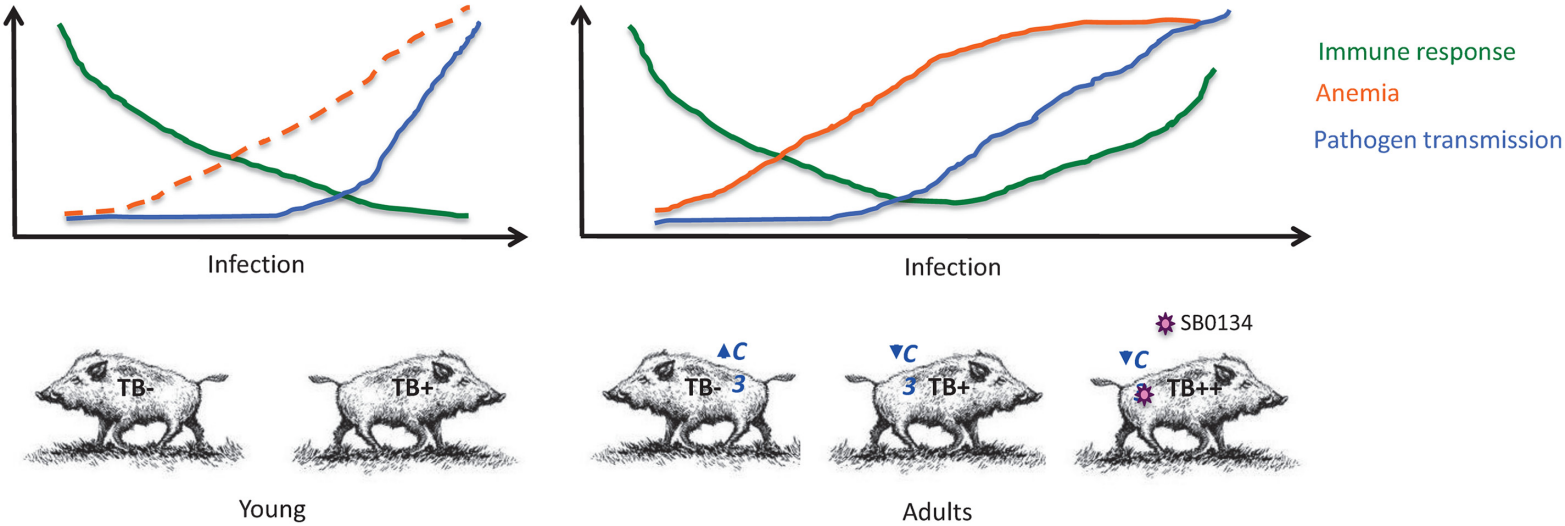
Host-mycobacteria interactions. Summary of the factors identified in this study with an impact on host-pathogen interactions. Mycobacteria manipulate host immune response by reducing the production of immune system proteins. However, as infection progresses, wild boar immune response recovers to limit pathogen multiplication and promote survival, facilitating pathogen transmission. Increased host survival in adult animals is probably associated with reduction in anemia progression. The upregulation of *C3* may be associated with protection in uninfected adult wild boar while higher lesion score in TB++ animals could be due to infection with *M*. *bovis* spoligotypes such as SB0134.

The results reported here suggested that *M*. *bovis* manipulates host immune response to facilitate infection in wild boar (Fig 8). As other intracellular bacteria, *M*. *bovis* manipulate host immune response by reducing the production of immune system proteins [26]. However, as infection progresses, wild boar immune response recover to limit pathogen multiplication and promote survival that also facilitates pathogen transmission (Fig 8). Adult TB++ wild boar with disseminated disease showed extensive macroscopic lesions with poor fibrotic containment of the granulomas and ulceration into the lumina of airways that facilitate pathogen transmission through aerogenous shedding of mycobacteria [13,42]. As previously reported for other obligate intracellular bacteria [43], host-mycobacteria interactions probably reflect a co-evolutionary process in which pathogens evolved mechanisms to subvert host response to establish infection but hosts also evolved mechanisms to limit pathogen infection and promote survival. Subsequently, mycobacteria benefit from host survival by increasing the probability for transmission to continue the life cycle. The reduction in anemia progression from TB+ to TB++ adult animals is probably associated with the increase in host survival (Fig 8).

These results also provided evidence to support the impact of host and pathogen derived genetic factors affecting pathogen infection and disease (Fig 8). The upregulation of *C3* in uninfected adult wild boar supported the role for this molecule in the protective mechanisms against TB [9, 44]. Additionally, some of the *M*. *bovis* spoligotypes such as SB0134 identified in adult TB++ animals may be associated with the high TB lesion score observed in these animals [24]. These results provide relevant information to develop tools to evaluate risks for TB caused by MTBC and for disease control in humans and animals.

## Supporting Information

S1 Fig Western blot analysis of wild boar recombinant proteins and mandibular lymph node protein extracts. To validate proteomics results, differentially represented *S*. *scrofa* immune system proteins S100A9, LTF and PGLYRP1 were produced in *E*. *coli* and used to generate rabbit antibodies. (A) Recombinant proteins (arrows) and lymph node protein extracts were separated in a 12% SDS-polyacrylamide gel. (B) Western blot analysis of recombinant proteins (arrows) with rabbit polyclonal antibodies. Some of the bands reacting with the antibodies with lower or higher molecular weight than the recombinant proteins likely correspond to degradation and polymerization products, respectively. (C-E) Western blot analysis of 15 μg of total proteins from individual wild boar mandibular lymph nodes (young TB-, N = 5; young TB+, N = 9; adult TB-, N = 4; adult TB+, N = 5; adult TB++, N = 5). Rabbit polyclonal antibodies against the recombinant immune system proteins and the ribosomal protein RPS14 included as control for normalization were used in Western blot analysis. The intensity of protein bands corresponding to test and control proteins were determined in the Western blot membrane by densitometric analysis. Abbreviation: MW, molecular weight markers.
(PDF)

S2 Fig Results of targeted proteomics in experimentally infected wild boar. To provide additional support for the results obtained in naturally infected animals, tonsils from wild boar experimentally infected with *M*. *bovis* under controlled conditions were used to characterize the levels of differentially represented immune system proteins C3S7K6, F1RRP1, Q6YT39, Q8SPA3, I3LNT1 and F1RM24 by targeted proteomics. The results were compared between naturally infected and experimentally infected TB+ and TB++ animals.
(PDF)

S1 Table Proteomics results in naturally infected wild boar. *S*. *scrofa* proteins identified with FDR<0.01 and at least two PSMs per protein in at least one of the analyzed samples, BP annotation and data quantitation.
(XLSX)

S2 Table Sequence of oligonucleotide primers used for gene cloning and real-time RT-PCR. (PDF)

S3 Table Proteomics results in experimentally infected wild boar. *S*. *scrofa* proteins identified with FDR<0.01 and at least two PSMs per protein in at least one of the analyzed samples and the *S*. *scrofa* proteins analyzed with FDR<0.05 in targeted proteomics.
(XLSX)
