# Supplementary material for: Comparative Proteomics Identifies Host Immune System Proteins Affected by Infection with Mycobacterium bovis
Source: PLoS Negl Trop Dis. 2016 Mar 30;10(3):e0004541. doi: 10.1371/journal.pntd.0004541 (PMC4814110; doi:10.1371/journal.pntd.0004541)
Supplement: S1 Fig — To validate proteomics results, differentially represented S. scrofa immune system proteins S100A9, LTF and PGLYRP1 were produced in E. coli and used to generate rabbit antibodies. (A) Recombinant proteins (arrows) and lymph node protein extracts were separated in a 12% SDS-polyacrylamide gel. (B) Western blot analysis of recombinant proteins (arrows) with rabbit polyclonal antibodies. Some of the bands reacting with the antibodies with lower or higher molecular weight than the recombinant proteins likely correspond to degradation and polymerization products, respectively. (C-E) Western blot analysis of 15 μg of total proteins from individual wild boar mandibular lymph nodes (young TB-, N = 5; young TB+, N = 9; adult TB-, N = 4; adult TB+, N = 5; adult TB++, N = 5). Rabbit polyclonal antibodies against the recombinant immune system proteins and the ribosomal protein RPS14 included as control for normalization were used in Western blot analysis. The intensity of protein bands corresponding to test and control proteins were determined in the Western blot membrane by densitometric analysis. Abbreviation: MW, molecular weight markers. (PDF) [file pntd.0004541.s001.pdf]

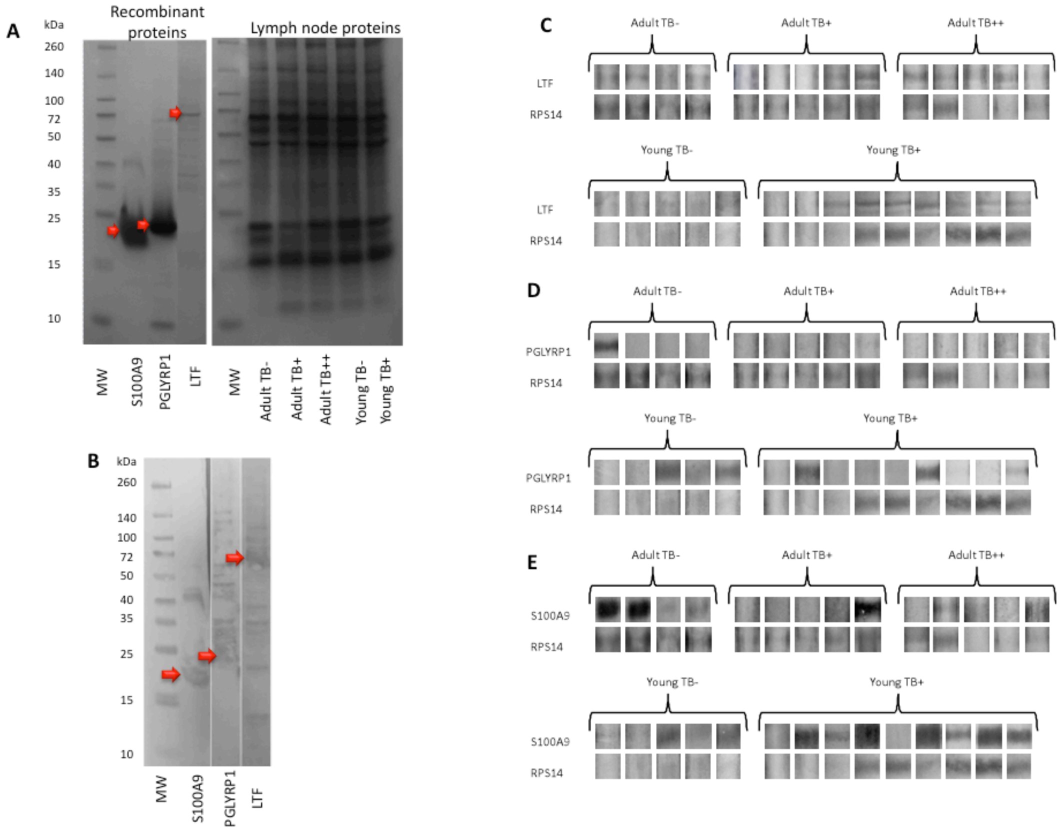

S1 Figure. Western blot analysis of wild boar recombinant proteins and mandibular lymph node protein extracts. To validate proteomics results, differentially represented *S. scrofa* immune system proteins S100A9, LTF and PGLYRP1 were produced in *E. coli* and used to generate rabbit antibodies. (A) Recombinant proteins (arrows) and lymph node protein extracts were separated in a 12% SDS-polyacrylamide gel. (B) Western blot analysis of recombinant proteins (arrows) with rabbit polyclonal antibodies. Some of the bands reacting with the antibodies with lower or higher molecular weight than the recombinant proteins likely correspond to degradation and polymerization products, respectively. (C-E) Western blot analysis of 15 µg of total proteins from individual wild boar mandibular lymph nodes (young TB-, N=5; young TB+, N=9; adult TB-, N=4; adult TB+, N=5; adult TB++, N=5). Rabbit polyclonal antibodies against the recombinant immune system proteins and the ribosomal protein RPS14 included as control for normalization were used in Western blot analysis. The intensity of protein bands corresponding to test and control proteins were determined in the Western blot membrane by densitometric analysis. Abbreviation: MW, molecular weight markers.
