# Supplementary material for: Comparative Proteomics Identifies Host Immune System Proteins Affected by Infection with Mycobacterium bovis
Source: PLoS Negl Trop Dis. 2016 Mar 30;10(3):e0004541. doi: 10.1371/journal.pntd.0004541 (PMC4814110; doi:10.1371/journal.pntd.0004541)
Supplement: S2 Fig — To provide additional support for the results obtained in naturally infected animals, tonsils from wild boar experimentally infected with M. bovis under controlled conditions were used to characterize the levels of differentially represented immune system proteins C3S7K6, F1RRP1, Q6YT39, Q8SPA3, I3LNT1 and F1RM24 by targeted proteomics. The results were compared between naturally infected and experimentally infected TB+ and TB++ animals. (PDF) [file pntd.0004541.s002.pdf]

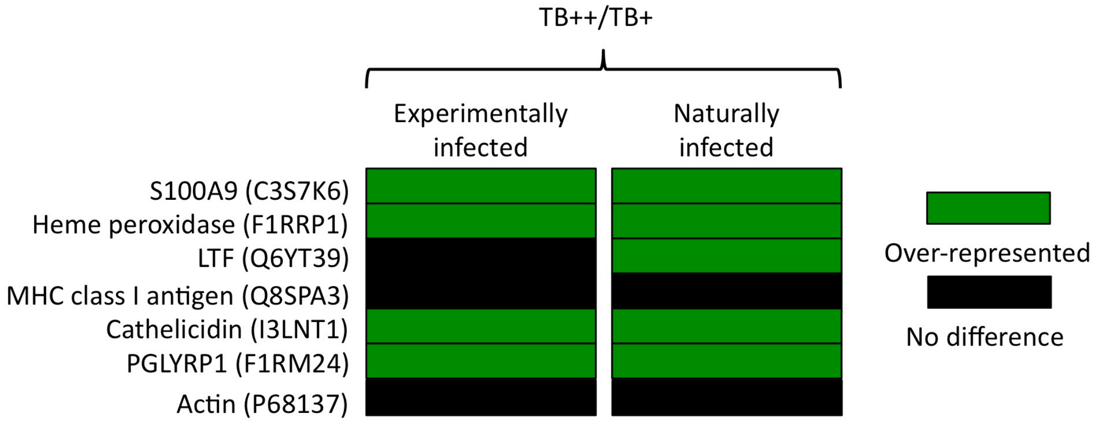

S2 Figure. Results of targeted proteomics in experimentally infected wild boar. To provide additional support for the results obtained in naturally infected animals, tonsils from wild boar experimentally infected with *M. bovis* under controlled conditions were used to characterize the levels of differentially represented immune system proteins C3S7K6, F1RRP1, Q6YT39, Q8SPA3, I3LNT1 and F1RM24 by targeted proteomics. The results were compared between naturally infected and experimentally infected TB+ and TB++ animals.
