# Supplementary material for: Comparative Proteomics Identifies Host Immune System Proteins Affected by Infection with Mycobacterium bovis
Source: PLoS Negl Trop Dis. 2016 Mar 30;10(3):e0004541. doi: 10.1371/journal.pntd.0004541 (PMC4814110; doi:10.1371/journal.pntd.0004541)
Supplement: S2 Table — (PDF) [file pntd.0004541.s004.pdf]

S2 Table. Sequence of oligonucleotide primers used for gene cloning and real-time RT-PCR.

GenBank accession number	Gene	Primer sequences (5'- 3')	Annealing conditions	Amplicon size (bp)
		Gene cloning		
AY306198.1	*Lactotransferrin* (*LTF*)	SscrofaTO-LTF-F: CACCATGAAGCTCTTCATCCCCGC	58°C, 120 sec	2115
		SscrofaTO-LTF-R: T CCTCATCATGAAGGCACAGG		
NM_001001260.1	*Peptidoglycan recognition protein 1* (*PGLYRP1*)	SscrofaTO-PGLYRP1-F: CACCATGGCCCGCAGCTGCGCGCT	62°C, 30 sec	584
		SscrofaTO-PGLYRP1-R: GGCGCGGTAGTGTGGCCATT		
NM_001177906.1	*Calcium binding protein A9* (*S100A9*)	SscrofaTO-S100A9-F: CACCATGGCGGACCAAATGTCGCA	60°C, 30 sec	432
		SscrofaTO-S100A9-R: GTGGCTGTGGCCATGGCCGT		
		Real time RT-PCR		
AY306198.1	*Lactotransferrin* (*LTF*)	Sscrofa-LTF-F: TGTCAGCTGTGCATAGGGAA	56°C, 30 sec	173
		Sscrofa-LTF-R: TATTTGTCCCGGTCAGCCTT		
NM_001001260.1	*Peptidoglycan recognition protein 1* (*PGLYRP1*)	Sscrofa_PGLYRP1-F: GAGGTCCGGCAAACTGCAT	56°C, 30 sec	245
		Sscrofa_PGLYRP1-R:		

		ATACACAAGCCCGTCTTCTC		
NM_001177906.1	*Calcium binding protein A9* (*S100A9*)	Sscrofa_S100A9-F: GCCCGCTCCTTGCTGTCCAA	56°C, 30 sec	250
		Sscrofa_S100A9-R: TCCAGGATGTGGTTTATGGC		
